# Supplementary material for: Compositionality in the semantic network: a model-driven representational similarity analysis
Source: Cereb Cortex. 2025 Sep 10;35(8):bhaf246. doi: 10.1093/cercor/bhaf246 (PMC12421894; doi:10.1093/cercor/bhaf246)
Supplement: Supplementary_materials_bhaf246 [file supplementary_materials_bhaf246.docx]

**Compositionality in the semantic network: A model-driven representational similarity analysis**

Marco Ciapparelli^1*^, Marco Marelli^1,2^, William Graves^3^, Carlo Reverberi^1,2^

**Authors affiliation and email address:**

^1^Department of Psychology, University of Milano-Bicocca, Milan, Italy

^2^NeuroMI, Milan Center for Neuroscience, Milan, Italy

^3^Department of Psychology, Rutgers University, Newark, New Jersey, USA

* Corresponding author; Mailing address: P.zza dell'Ateneo Nuovo 1, Milan, Italy; e-mail: [m.ciapparelli1@campus.unimib.it](mailto:m.ciapparelli1@campus.unimib.it)

**Supplementary materials**

**Supplementary S1: Comparison of beta series estimation methods**

Our staggered estimation approach is inspired by the Least-Squares Separate (LS-S) method (Mumford et al., 2012), which consists in running, for each trial, a separate GLM where the target trial is modeled by a regressor and all other trials are aggregated and modeled by a single nuisance regressor. Because we needed to estimate hundreds of trials for each subject in a sample of 85 subjects, we adopted a staggered approach to reduce the computational and memory cost of beta estimation. As the LS-S is a popular technique to deal with autocorrelation in condition-rich designs (Prince et al. 2022), we show – following reviewers’ suggestions – how the beta maps estimated with our staggered approach align with those obtained with the LS-S method.

First, we re-estimated all beta coefficients following the LS-S approach, so that each trial (i.e., each two-word phrase stimulus) was described by a vector of betas estimated i) with our staggered approach, ii) with LS-S. We focused on the beta maps of the five ROIs considered, namely the LATL, LAG, RAG, LIFG, and the general semantic network as defined in our searchlight RSA (i.e., the intersection of a mask excluding all voxels missing from at least 9 subjects and a mask of the general semantic network peaks from Jackson, 2021). For each ROI, we compared beta estimates in two ways, looking at both first- and second-order similarities. First, we computed the Pearson correlation coefficients between each pair of beta vectors, computed their mean and median value across all pairs (i.e., trials) within each subject, and computed the mean and standard deviation of those mean and median values across all 85 subjects (Table S1). The two methods are highly aligned, with the lowest alignment being $r=0.944$ (mean of means) observed in the LATL. Second, we conducted representational similarity analyses (RSAs) between activation matrices. Specifically, for each beta estimation approach, we defined a representational dissimilarity matrix coding for the Mahalanobis distance among all pairs of beta vectors, excluding stimuli falling in outlier volumes identified with TsDiffAna (see section 2.2.3). For the semantic network, because its number of voxels exceeded the number of trials, the Mahalanobis distance among beta vectors could not be computed for this ROI, and the Euclidean distance was used instead. Finally, we computed the Spearman correlation coefficient between the upper-triangular portion of representational dissimilarity matrices. Table S1 reports the mean and standard deviation of these Spearman values across subjects for each ROI. Figure S1 complements the table with boxplots. Again, the two beta estimation methods lead to high representational similarities, the lowest being $r=0.907$ in the general semantic network.

*Table S1:* Results of the comparison of beta maps estimated with the staggered or LS-S approaches. Each cell reports the mean and standard deviation (in parentheses) of a given similarity metric.

| **similarity metric** | | **LATL** | **LAG** | **RAG** | **LIFG** | **Semantic network** |
| --- | --- | --- | --- | --- | --- | --- |
| **first-order** | mean | 0.944 (0.020) | 0.948 (0.019) | 0.948 (0.021) | 0.948 (0.020) | 0.950 (0.019) |
|  | median | 0.960 (0.015) | 0.962 (0.013) | 0.961 (0.014) | 0.960 (0.013) | 0.959 (0.014) |
| **second-order (RSA)** | | 0.920 (0.038) | 0.913 (0.039) | 0.912 (0.041) | 0.922 (0.039) | 0.907 (0.052) |


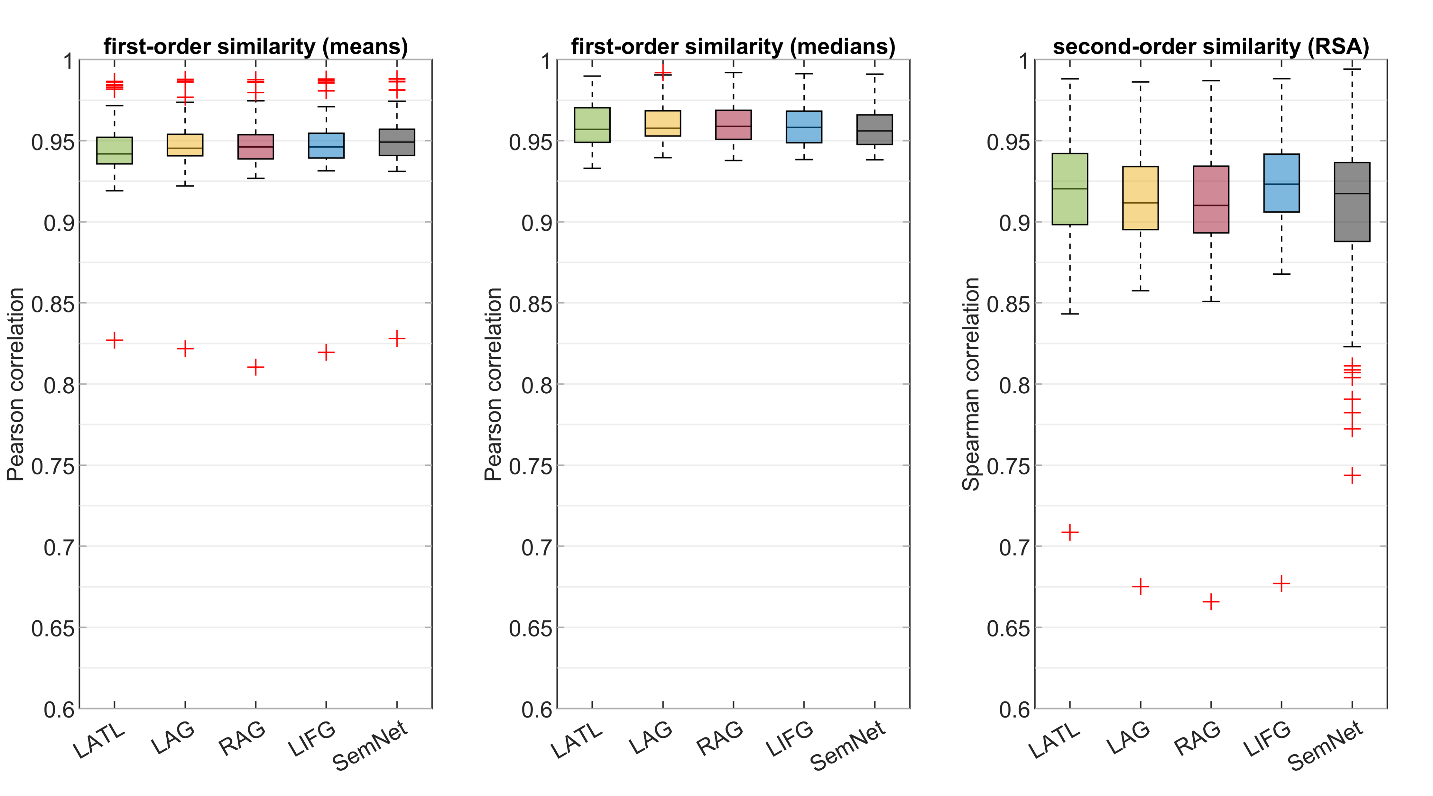


Figure S1: Boxplots illustrating the similarity of beta maps estimated with the staggered or LS-S approaches.

**Supplementary S2: ROI coordinates**

*Table S2:* Core ROI coordinates. For each core ROI, the table reports the mean MNI coordinates computed based on the peak activations reported in relevant studies (listed in the rightmost column).

| **region** | **x** | **y** | **z** | **studies** |
| --- | --- | --- | --- | --- |
| left anterior temporal lobe (LATL) | –48.1 | 0.1 | –24.5 | (Wang et al., 2020) |
|  |  |  |  | (Graessner et al., 2021) |
|  |  |  |  | (Forgács et al., 2012) |
|  |  |  |  | (Feng et al., 2015) |
|  |  |  |  | (Molinaro et al., 2015) |
|  |  |  |  |  |
| Left angular gyrus  (LAG) | –40.2 | –66.6 | 36.9 | (Wang et al., 2020) |
|  |  |  |  | (Ford et al., 2010) |
|  |  |  |  | (Schell et al., 2017) |
|  |  |  |  | (Graessner et al., 2021) |
|  |  |  |  | (Forgács et al., 2012) |
|  |  |  |  | (Zhang et al., 2022) |
|  |  |  |  |  |
| Right angular gyrus (RAG) | 46.9 | –62.3 | 35.2 | (Graessner et al., 2021) |
|  |  |  |  | (Graves et al., 2010) |
|  |  |  |  | (Ford et al., 2010) |
|  |  |  |  | (Forgács et al., 2012) |
|  |  |  |  |  |
| Left inferior frontal gyrus (LIFG) | –47.4 | 21.3 | 7.7 | (Wang et al., 2020) |
|  |  |  |  | (Schell et al., 2017) |
|  |  |  |  | (Graessner et al., 2021) |
|  |  |  |  | (Molinaro et al., 2015) |
|  |  |  |  | (Graves et al., 2010) |
|  |  |  |  | (Ford et al., 2010) |
|  |  |  |  | (Forgács et al., 2012) |
|  |  |  |  | (Feng et al., 2015) |

**Supplementary S3: Tables of results**

*Table S3:* Core ROI results. Right-tailed Wilcoxon signed-rank test results for all models. Test statistics were computed over the Spearman correlation coefficients (i.e., subject-level RSA results) for all subjects and all studies (N = 85). RSAs were performed with Spearman correlations; partial RSAs controlling for confounding models were performed with partial Spearman correlations (models controlled in such a manner are specified with the *p* subscript). The table reports Z scores and uncorrected *p-*values; results significant after Bonferroni correction (adjusted $\alpha=.0$125) are reported in **bold.**

| **RDM predictor** | **LATL** | | **LAG** | | **RAG** | | **LIFG** | |
| --- | --- | --- | --- | --- | --- | --- | --- | --- |
|  | $Z$ | $p$ | $Z$ | $p$ | $Z$ | $p$ | $Z$ | $p$ |
| $run$ | **7.186** | **<.001** | **7.808** | **<.001** | **7.554** | **<.001** | **7.195** | **<.001** |
| $Levenshtein$  $distance$ | -0.272 | .607 | -0.911 | .819 | 0.438 | .331 | -0.311 | .622 |
| $w_{1} frequency$ | -0.583 | .720 | -0.074 | .530 | -0.245 | .597 | -0.193 | .576 |
| $w_{2} frequency$ | -0.692 | .756 | 0.232 | .408 | 1.726 | .042 | -0.596 | .724 |
| $concreteness$ | 0.355 | .361 | 0.079 | .469 | -0.197 | .578 | 0.241 | .405 |
| ${w_{1}}_{p}$ | 1.503 | .066 | 1.209 | .113 | 0.508 | .306 | *2.138* | *.016* |
| $w_{2_{p}}$ | 0.618 | .268 | **2.892** | **.002** | 0.140 | .444 | 1.481 | .069 |
| $additive_{p}$ | 1.393 | .082 | **3.558** | **<.001** | 1.315 | .094 | *1.998* | *.023* |
| $multiplicative_{p}$ | *2.090* | *.018* | 1.323 | .093 | 0.539 | .295 | **2.300** | **.011** |
| $CAOSS_{p}$ | 0.206 | .418 | 0.500 | .309 | 0.364 | .358 | 0.018 | .493 |

*Table S4:* Peak activations for the searchlight RSA in the semantic network. Coordinates [x, y, z] in Montreal Neurological Institute (MNI) space and selection of cluster maxima according to SPM12. Searchlight RSA was performed with partial Spearman correlations controlling semantic and compositional semantic models for baseline models.

| **model** | **Cluster *p* (FWE)** | **Cluster size** | **Peak Z** | **MNI** | | |
| --- | --- | --- | --- | --- | --- | --- |
|  | | | | | | |
| $multiplicative$ | .0004 | 452 | 3.74 | –64 | –12 | 2 |
|  |  |  | 3.60 | –60 | –22 | 2 |
|  |  |  | 3.42 | –66 | –24 | –8 |
|  | .030 | 186 | 3.55 | –58 | 24 | 8 |
|  |  |  | 3.47 | –44 | 26 | 8 |
|  |  |  | 3.20 | –52 | 26 | 0 |
